# Sequence based typing of HLA-A and B Exons-2 and -3 in a HIV-positive native community with limited HLA diversity from the North of Argentina

**DOI:** 10.1186/1742-4690-9-S2-P164

**Published:** 2012-09-13

**Authors:** DA Dilernia, D Mónaco, R Coloccini, M Pando, M Quipildor, A Di Paolo, E Hunter, H Salomon

**Affiliations:** 1 Argentinean Reference Center for AIDS, Buenos Aires, Argentina; 2 Hospital San Vicente de Paul, San Ramón de la Nueva Oran, Argentina; 3 Emory Vaccine Center, Atlanta, GA, USA

## Background

We previously reported a limited diversity of HLA-class I alleles in two-digits typing studies of HIV-positive native populations from Oran, North of Argentina. In the present study we determine whether the restricted diversity observed at low-resolution reflects also a restricted genetic diversity in HLA peptide groove.

## Methods

We studied 65 HIV-positive patients whose HLA-A and -B genes were previously typed by SSOP technique. We set-up a sequence-based typing of the most prevalent alleles in Oran. HLA-A and B were initially PCR-amplified and exons-2 and -3 were sequenced. NCBI-SBT-Interpretation tool was used to confirm the two-digits typing with previous SSOP data. We designed a set of primers specifics for HLAs highly prevalent in Oran to achieve differential PCR-amplification of each allele in heterozygote patients. Phylogenetic analysis was used to assign exons-2 and -3 sequences to a high-resolution HLA group.

## Results

Our results show that for HLA-A alleles, 85.1% of A*02 are A*02:01:01:01, 96.7% of A*31 are A*31:01:02 and 92.8% of A*24 are A*24:02:01:01. In the case of A*68, 50% are A*68:01:02 and 31.2% are A*68:17. For HLA-B alleles, B*35 was diverse: B*35:01:01:01 (15.8%), B*35:04:01 (15.8%), B*35:05:01 (21.1%) and B*35:19 (21.1%). 43.8% of B*39-alleles were B*39:05:01 and 25% were B*39:03. 52.9% of B*48-alleles were B*48:01:01 and 35.3% were B*48:03:01. 62.5% of B*51 alleles were B*51:01:01. The mentioned alleles represent the 73.1% of HLA-A genetic diversity and the 45.4% of HLA-B. All the polimorphisms observed lead to non-synonymous changes.

## Conclusion

Our results show that two-digits typing of HLA-A usually corresponds with a specific allele in our population. For HLA-B alleles, observed within-subtype diversity was higher. The different protein sequence encoded by exons-2 and -3 may lead to different peptide specificities among alleles from the same HLA-B subtype that would be miss-classified as homogeneous in a low-resolution typing study.

